# Myocardial Perfusion Imaging After Severe COVID-19 Infection Demonstrates Regional Ischemia Rather Than Global Blood Flow Reduction

**DOI:** 10.3389/fcvm.2021.764599

**Published:** 2021-12-07

**Authors:** George D. Thornton, Abhishek Shetye, Dan S. Knight, Kris Knott, Jessica Artico, Hibba Kurdi, Souhad Yousef, Dimitra Antonakaki, Yousuf Razvi, Liza Chacko, James Brown, Rishi Patel, Kavitha Vimalesvaran, Andreas Seraphim, Rhodri Davies, Hui Xue, Tushar Kotecha, Robert Bell, Charlotte Manisty, Graham D. Cole, James C. Moon, Peter Kellman, Marianna Fontana, Thomas A. Treibel

**Affiliations:** ^1^Barts Heart Center, Barts Health NHS Trust, London, United Kingdom; ^2^Institute of Cardiovascular Science, University College, London, United Kingdom; ^3^Royal Free London NHS Foundation Trust, London, United Kingdom; ^4^Division of Medicine, National Amyloidosis Center, University College, London, United Kingdom; ^5^Imperial College Healthcare NHS Trust, London, United Kingdom; ^6^National Heart and Lung Institute, Imperial College London, London, United Kingdom; ^7^National Heart, Lung, and Blood Institute, National Institute of Health, Bethesda, MD, United States; ^8^Department of Cardiology, University College London Hospitals NHS Trust, London, United Kingdom

**Keywords:** cardiac MRI, perfusion, COVID-19, microvascular dysfunction, myocardial blood flow

## Abstract

**Background:** Acute myocardial damage is common in severe COVID-19. Post-mortem studies have implicated microvascular thrombosis, with cardiovascular magnetic resonance (CMR) demonstrating a high prevalence of myocardial infarction and myocarditis-like scar. The microcirculatory sequelae are incompletely characterized. Perfusion CMR can quantify the stress myocardial blood flow (MBF) and identify its association with infarction and myocarditis.

**Objectives:** To determine the impact of the severe hospitalized COVID-19 on global and regional myocardial perfusion in recovered patients.

**Methods:** A case-control study of previously hospitalized, troponin-positive COVID-19 patients was undertaken. The results were compared with a propensity-matched, pre-COVID chest pain cohort (referred for clinical CMR; angiography subsequently demonstrating unobstructed coronary arteries) and 27 healthy volunteers (HV). The analysis used visual assessment for the regional perfusion defects and AI-based segmentation to derive the global and regional stress and rest MBF.

**Results:** Ninety recovered post-COVID patients {median age 64 [interquartile range (IQR) 54–71] years, 83% male, 44% requiring the intensive care unit (ICU)} underwent adenosine-stress perfusion CMR at a median of 61 (IQR 29–146) days post-discharge. The mean left ventricular ejection fraction (LVEF) was 67 ± 10%; 10 (11%) with impaired LVEF. Fifty patients (56%) had late gadolinium enhancement (LGE); 15 (17%) had infarct-pattern, 31 (34%) had non-ischemic, and 4 (4.4%) had mixed pattern LGE. Thirty-two patients (36%) had adenosine-induced regional perfusion defects, 26 out of 32 with at least one segment without prior infarction. The global stress MBF in post-COVID patients was similar to the age-, sex- and co-morbidities of the matched controls (2.53 ± 0.77 vs. 2.52 ± 0.79 ml/g/min, *p* = *0.10*), though lower than HV (3.00 ± 0.76 ml/g/min, *p*< *0.01*).

**Conclusions:** After severe hospitalized COVID-19 infection, patients who attended clinical ischemia testing had little evidence of significant microvascular disease at 2 months post-discharge. The high prevalence of regional inducible ischemia and/or infarction (nearly 40%) may suggest that occult coronary disease is an important putative mechanism for troponin elevation in this cohort. This should be considered hypothesis-generating for future studies which combine ischemia and anatomical assessment.

## Introduction

Coronavirus disease 2019 caused by severe acute respiratory syndrome coronavirus 2 (SARS-CoV-2), disproportionally affects patients with cardiovascular risk factors. Myocardial injury, particularly seen in severe and hospitalized COVID-19 and evidenced by raised cardiac troponin, heralds worse outcomes (1, 2). However, the mechanisms of injury remain only partially understood, and the potential coronary microcirculatory sequelae remain incompletely characterized (3). Several ischemic and non-ischemic mechanisms have been proposed, including supply-demand mismatch (type-2 myocardial infarction) and microangiopathic thrombosis (4). Accumulating evidence suggests that the vascular endothelium plays a critical role in the pathogenesis of severe COVID-19 as a nidus for both pro-coagulant and inflammatory dysregulation and may offer a unifying pathway through which all these sequelae may occur (5, 6). Autopsy results have shown microthrombi to be associated with myocyte necrosis (7). The implications for survivors and the potential long-term effects on coronary microcirculation remain unknown. Cardiovascular magnetic resonance can determine not only myocardial function, remodeling, and scar burden, but also quantify the stress myocardial blood flow (MBF), which has been validated invasively and against ^13^N–NH_3_ PET (8–10). A recent pilot study of *n* = 22 recovered COVID-19 patients used coronary sinus flow by cardiovascular magnetic resonance (CMR) to evaluate the myocardial perfusion found with significantly lower myocardial perfusion reserve (MPR) compared with an unmatched cohort of health controls and values similar to a cohort with hypertrophic cardiomyopathy (HCM) (11). We aimed to further evaluate the pattern of stress MBF in recovered COVID-19 patients.

## Methods

### Patient Population

#### COVID Cohort

Patients clinically referred for adenosine stress CMR following their admission for COVID-19 to three CMR centers (Royal Free London NHS Foundation Trust [RFH], Imperial College Healthcare NHS Trust [Imperial], and University College London Hospital [UCLH] NHS Foundation Trust) were recruited for the study at the time of their CMR. We included patients with stress perfusion imaging from our recently published multicenter study (12) to the overall cohort and performed dedicated quantitation of MBF. The patients had a diagnosis of COVID-19 made either by (i) a positive combined oro/nasopharyngeal throat swab or tracheal aspirate for SARS-CoV-2 by reverse-transcriptase-polymerase-chain-reaction (RT-PCR), or (ii) a negative swab for SARS-CoV-2 but with a triad of symptoms of viral illness (such as one or more of cough, fever, and myalgia), typical blood biomarkers (such as new lymphopenia, high d-dimer, high ferritin, and elevated liver transaminases), and reported findings of at least probable likelihood of COVID-19 infection on chest radiograph or CT (12). Indications for CMR included positive troponin during hospital admission (*n* = 85; hsTnT >14ng/L for RFH and UCLH; hsTnI >14ng/L for females and >34ng/L for males for Imperial) or persistent symptoms (*n* = 5; chest pain or shortness of breath) in COVID-19 recovery. The exclusion criteria included patient refusal, severe renal impairment (estimated glomerular filtration rate (eGFR) <30 mL/min/m^2^, if local hospital policy excluded these patients), pregnancy, medical unsuitability assessed by the referring clinician (including severe co-morbid disease and/or frailty in which it was felt that the information acquired would be unlikely to alter clinical management), and standard CMR contraindications. Ethical approval was obtained from the West Midlands—Edgbaston Research Ethics Committee for the use of the clinical data of the patients for research purposes (RFH and Imperial sites; REC reference 20/WM/0208) and from the Joint University College London/University College London Hospitals Research Ethics Committee (UCLH site; REC reference 07/H0715/101).

### Control Cohort and Healthy Volunteers

#### Control Cohort

Patients referred for clinical adenosine stress CMR with contemporaneous invasive or CT coronary angiography (CTCA) without obstructive coronary disease between May 2016 and December 2019 (Pre-COVID) were recruited at two centers: Barts Heart Center (BHC) and Royal Free Hospital (RFH). Patients with significant coronary artery disease (diameter stenosis on coronary angiography >30%), previous coronary revascularization, infarct pattern scar, non-ischemic scar, or cardiomyopathy (hypertrophic, arrhythmogenic, dilated, amyloid) were excluded. The control cohort was propensity-matched to the COVID-19 patient cohort. A control group of known unobstructed coronary arteries was selected as the cleanest possible control, as the coronary status of the majority of the COVID cohort was unknown and thus could not be adjusted for.

#### Healthy Volunteers

Twenty-seven healthy volunteers {median age 33 [interquartile range (IQR) 30-42] years, 14[52%] male} were prospectively recruited and underwent adenosine stress CMR. These were individuals with no risk factors for coronary artery disease and were not taking any medication. The participants gave their written informed consent according to the local ethics applications. The control and healthy volunteer studies were approved by the National Health Service Research Ethics Committee (NHS REC) and Health Research Authority (HRA) and were conducted in accordance with the Declaration of Helsinki (Barts Bioresource - REC ID 14/EE/0007, Royal Free Hospital – REC ID 07/H0715/101).

### CMR Study Protocol

The CMR was performed in accordance with the local institutional and international infection control guidelines (13) on 1.5T scanners (Magnetom Aera, Siemens Healthcare, Erlangen, Germany). A standard CMR protocol including parametric mapping, adenosine stress perfusion, and post-contrast imaging was used (Supplementary Figure 1): standard long- (4-, 2-, 3-chamber) and short-axis cine images were performed with breath-hold or real-time imaging, as needed. Native T1 and T2 mapping were performed in at least one long axis and one mid-ventricular short-axis view. The T1 mapping used the modified Look-Locker inversion recovery (MOLLI) sequence after regional shimming with 5s(3s)3s sampling (14). The T2 mapping used single-shot T2-prepared images acquired at multiple echo times (TE) (15). Following the application of 0.1 mmol/kg gadoterate meglumine (Royal Free and UCLH) or gadobutrol (Imperial), bright-blood late gadolinium enhancement (LGE) images were acquired using respiratory motion-corrected sequences with magnitude and phase-sensitive inversion recovery reconstructions (16). The patients underwent adenosine stress perfusion after refraining from caffeine for at least 24 h. Three short-axis views were acquired during the adenosine hyperemia (140 mcg/kg/min adenosine for 4 min with two further minutes at 175 mcg/kg/min if needed). The acquisition was for 60 heartbeats using a 0.05 mmol/kg gadolinium bolus administered at 4 mL/s followed by a 20-mL 0.9% saline flush. Perfusion maps (three short-axis slices per patient) were generated automatically and inline at the time of the scan as described by Kellman et al. (8). The perfusion is quantified for each pixel of the myocardium, each pixel encoding the MBF. The automated segmentation of the left ventricle (LV) using artificial intelligence (AI) techniques enables the calculation of global and segmental mean blood flow (in ml/g/min) as previously described (17).

### CMR Post-processing

The CMR studies were analyzed offline using CVI42 5.12.1 (Circle Cardiovascular Imaging, Calgary, Canada). All the cines, maps, first-pass perfusion images/maps, and early/late gadolinium enhancement images were analyzed by experienced observers blinded to the coronary data. When calculating the ventricular volumes and mass, the trabeculations and papillary muscles were included in the myocardial mass. For the patients with visual evidence of LGE, the endo and epicardial contours (10% offset) were drawn and automatically divided into six segments, a 3 SD approach was taken, and those without visual LGE were marked as zero. Limited right ventricular (RV) insertion point LGE was not included as an abnormal LGE finding. The native T1 and T2 relaxation times were measured within the myocardial septum in the basal inferoseptum on motion-corrected quantitative maps and away from any areas of LGE (remote myocardium). Where a non-infarct pattern LGE was seen, the native T1 and T2 in the same region were measured. Phantom quality assurance was performed to ensure the stability and inter-site comparability of T1 as reported previously (12). The perfusion defects were determined upon visual inspection of the first-pass perfusion images and corroborated with a visual inspection of the quantitative perfusion maps. The perfusion defects were compared against LGE imaging to match the perfusion defects to the areas of infarct-pattern LGE. The perfusion defects were defined as “unmatched” if it occurs in the absence of infarct-pattern LGE or if extending beyond the area of late enhancement. Quantitative myocardial perfusion maps were generated automatically, in-line without user input, however, all the studies were visually inspected for motion correction (MOCO) quality, artifact and graphically (review of the arterial input function), see Supplementary Figure 2. Where significant issues were identified, the maps were reviewed and reconstructed where possible.

### Statistical Analysis

Statistical analysis was performed using R Studio version 1.3 (R Studio, Boston, Massachusetts, Unites States). The data is presented as mean± SD and were normally distributed and median (25th−75th quartile) otherwise. The categorical variables are presented as absolute values and percentages. Comparison of data was performed using an unpaired *t*-test (two groups) or one-way ANOVA (three groups), and non-normally distributed the data using Mann–Whitney/Kruskal–Wallis tests as appropriate. The categorical data were compared using the Chi-Squared test or Fisher's Exact test, where appropriate. Propensity score matching was used to adjust for the imbalance in the COVID and control cohorts as follows. Logistic regression was used to predict the propensity score for either the control or the COVID cohort. The model was selected based on the characteristics of the patient that felt to be most clinically relevant to the study hypothesis, i.e. risk factors for cardiovascular/coronary artery disease. The balance of prognostic factors was inspected *via* standardized mean differences. Matching was performed without replacement using a “greedy” algorithm (nearest neighbor). Linear regression was performed to identify the multivariable predictors of global stress MBF in the COVID and control cohorts. Clinically relevant variables and those found to have a significant univariable association with global stress MBF were included. The model was then pruned using the backward stepwise selection by Akaike Information Criterion (AIC). All values with *p* < 0.05 were considered statistically significant.

## Results

### Clinical Characteristics

#### COVID Cohort

In this study, 90 patients were included (49 RFH, 37 UCLH, 4 Imperial)—see Supplementary Figure 3. Out of the 90 patients, 82 (91%) patients had a positive COVID-19 PCR test. The median age was 64 (IQR 54–71) years, 75(83%) male. Comorbidities were diabetes mellitus 29 (32%), hypertension 43 (48%), dyslipidemia 34 (38%), smoking history 25 (28%), and previous coronary revascularization 5 (5.6%). The median stay was 12 days (IQR 7-28), of which 40 out of 90 (44%) had been admitted to the intensive care unit (ICU). The median time from the discharge to CMR was 61 days (IQR 29-146). The median peak troponin T concentration (excluding Imperial patients [*n* = 4] who had troponin I assay) was 27 ng/ml (IQR 19–70), the peak NT-proBNP was 314 pg/ml (IQR 102–878), and the peak D-dimer was 3,444 ng/ml (IQR 1,217–10,092).

#### Controls

The control cohort was propensity-matched for age, sex, hypertension, type-2 diabetes, and smoking history (*p* for all >0.05). The mean standardized differences before and after matching are presented in Supplementary Table 1.

### Cardiovascular Magnetic Resonance

**Figure 1:**
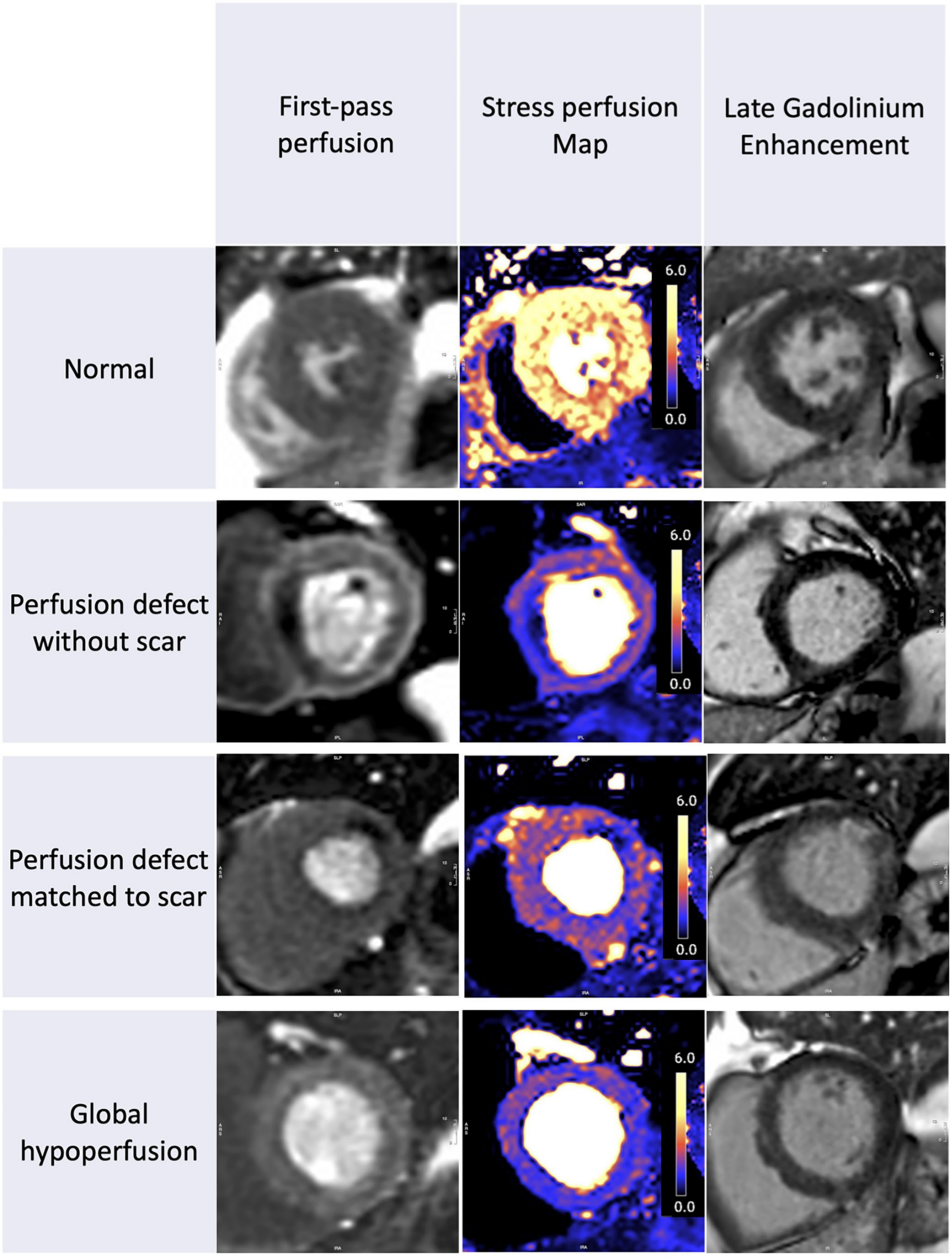
Stress perfusion and scar in recovered COVID-19 patients. The spectrum of perfusion abnormalities. From left to right we show the first-pass perfusion images, quantitative stress perfusion maps, and free breathing-phase sensitive inversion recovery and motion corrected late gadolinium enhancement images (PSIR MOCO LGE). Patient 1: Normal. Patient 2: Regional ischemia without LGE. Patient 3: Regional ischemia with infarct late gadolinium enhancement (LGE). Patient 4: Global hypoperfusion without visual perfusion defects and no significant LGE.

The patient characteristics and CMR findings are summarized in Table 1, the case examples are shown in Figure 1.

**Table 1:** Baseline characteristics.

**Characteristic**	**COVID** ***n* = 90^**a**^**	**Controls** ***n* = 90^**a**^**	**HV** ***n* = 27^**a**^**	***p*-value (COVID vs control)^**b**^**	***p*-value (All groups)^**b**^**
Age	64 (54, 71)	60 (49, 68)	33 (30, 42)	0.074	**<0.001**
Sex				0.85	**0.002**
Female	15 (17%)	17 (19%)	13 (48%)		
Male	75 (83%)	73 (81%)	14 (52%)		
Type 2 diabetes	29 (32%)	26 (29%)	0	0.75	**0.003**
Hypertension	43 (48%)	48 (53%)	0	0.55	**<0.001**
Dyslipidemia	34 (38%)	45 (50%)	0	0.13	**<0.001**
Prior history of CAD	23 (26%)	0	0	**<0.001**	**<0.001**
PCI/CABG	5 (5.6%)	0	0	>0.99	>0.99
Smoker	25 (28%)	27 (30%)	0	0.87	**0.005**
ICU Admission	40 (44%)	–	–		
Troponin (ng/L)^*^	27 (19, 70)	–	–		
NT-proBNP (pg/ml)	314 (102, 878)	–	–		
D-dimer (ng/ml)	3,444 (1,217, 10,092)	–	–		
CRP (mg/L)	223 (141, 344)	–	–		
LV EDV (ml)	130 (112, 147)	142 (119, 164)	147 (127, 156)	0.075	0.063
LV mass (g)	126 (109, 144)	110 (94, 132)	97 (86, 114)	**<0.001**	**<0.001**
LVEF (%)	67 (10)	67 (8)	65 (4)	0.91	0.81
RVEF (%)	59 (8)	–	–		
Native T1 (ms)	1,032 (1,008, 1,061)	–	–		
T2 (ms)	46 (45, 47)	–	–		
ECV (%)	26 (23, 29)	–	–		
LGE Present	50 (56%)	0	0	**<0.001**	**<0.001**
Infarct pattern LGE	15 (17%)	0	0	**<0.001**	**<0.001**
Non-ischemic LGE	31 (34%)	0	0	**<0.001**	**<0.001**
Mixed Pattern LGE	4 (4.4%)	0	0	0.12	0.11

a *Mean (SD); n (%); Median (IQR)*,

b *One-way ANOVA; chi-square test of independence; Fisher's exact test; Kruskal-Wallis test*.

* *Including patients with troponin T only (excluding four patients from Imperial College Healthcare NHS Trust (Imperial) who had troponin I assay). p-values reaching statistical significance (p < 0.05) are highlighted in bold*.

#### Cardiac Function and Myocardial Tissue Characterization

In the COVID cohort, the LV ejection fraction (EF) was 67 ± 10% and the RV EF was 59 ± 8% with 10 (11%) patients with LV systolic impairment and 6 (7%) patients with RV impairment. There was no difference in LVEF compared with the propensity-matched cohort or healthy volunteers (*p* = 0.81).

**Figure 2:**
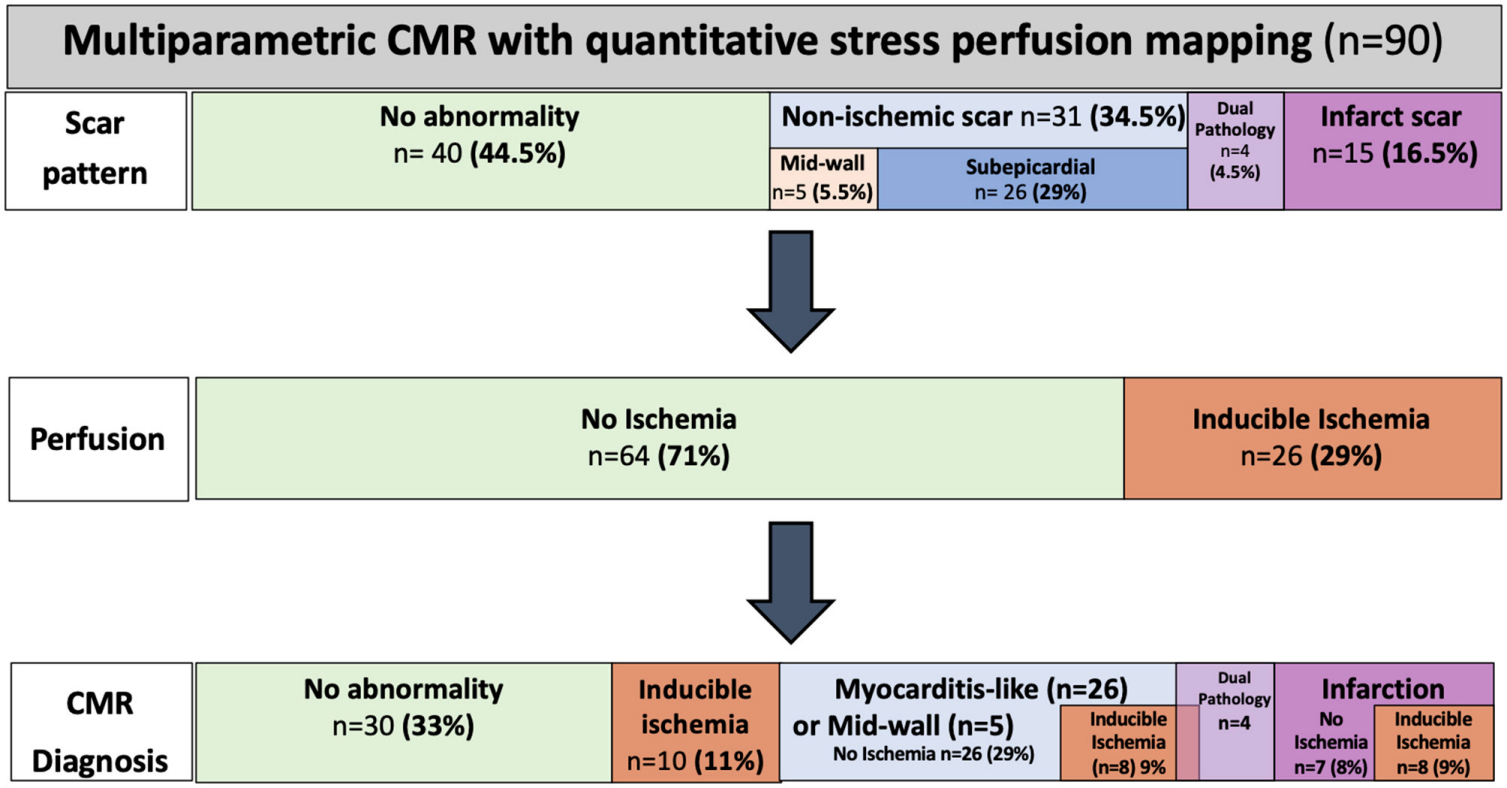
Cardiovascular magnetic resonance (CMR) findings and diagnosis by perfusion CMR. The CMR scar patterns, prevalence of ischemia, and diagnosis across the COVID-19 cohort.

In the post-COVID cohort, 50 (56%) patients had evidence of myocardial scar wherein 15 out of 90 (17%) patients had an infarct pattern, 31 out of 90 (34%) had a non-infarct pattern LGE, and four out of 90 (4.4%) patients had a dual/mixed pattern LGE. There was sub-epicardial myocarditis-like LGE in 26 out of the 90 (29%) patients, mid-wall LGE in 5, and four patients had both infarct and non-infarct pattern LGE. The control cohort was specifically selected for the absence of LGE; none of the healthy volunteers had LGE. For the breakdown of the scar patterns and CMR findings, see Figure 2.

The median T1 was 1,032ms (IQR 1,008–1,061ms) and the median T2 was 46 (IQR 45-47ms) in the basal inferoseptum, remote to the LGE. Thirteen patients had evidence of ongoing myocardial edema (T2 >50ms) in the regions coinciding with LGE. The normal ranges for T1 and T2 were from the data from the healthy volunteers from our recent phantom controlled work (native T1 1008 +/− 35ms, T2 48 +/− 2ms)(12).

#### Quantitative Stress MBF

##### Global Perfusion

**Figure 3:**
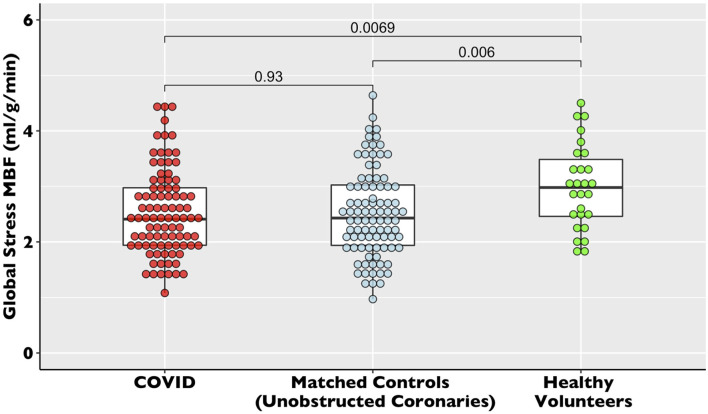
Global stress myocardial blood flow (MBF) in recovered post-COVID19 patients vs. propensity-matched controls and healthy volunteers. The dot plot of global stress myocardial blood flow for the COVID-19, control, and healthy volunteer cohorts. The data are presented with accompanying box plots.

**Figure 4:**
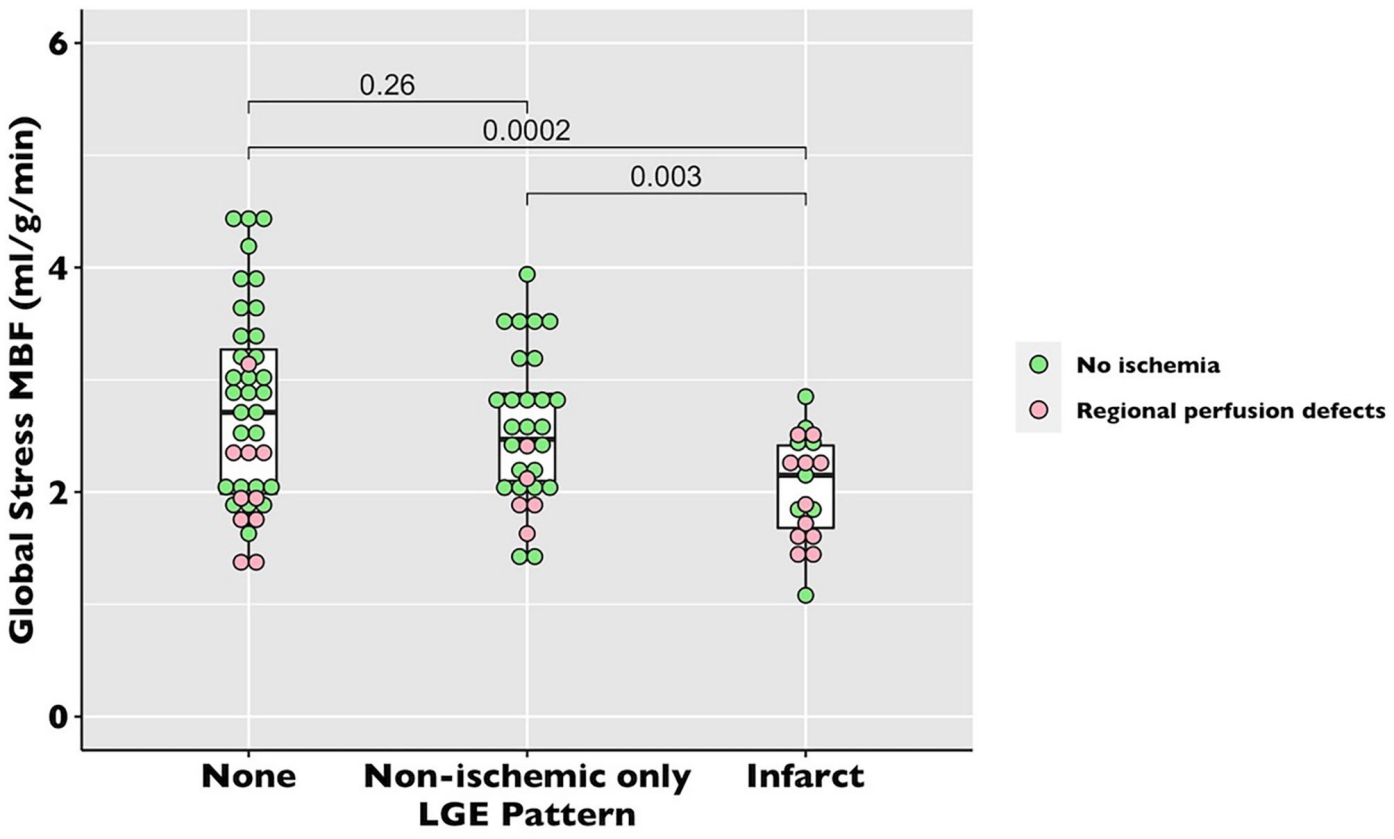
Global stress MBF in recovered post-COVID-19 patients by late gadolinium enhancement (LGE) pattern. The dot plot of global stress MBF by perfusion pattern. Each dot represents a patient. The pink dots represent the patients with unmatched regional perfusion defects by visual assessment. The green dots represent the patients with no regional perfusion defects.

There was no difference in global stress MBF (2.53 ± 0.77 vs. 2.52 ± 0.79 ml/g/min, *p* = *0.10*) in the COVID patients vs. propensity-matched controls. The healthy volunteers had higher stress MBF than either the COVID or control volunteers (sMBF 3.0 ± 0.76 ml/g/min, *p* = *0.01*) (Table 2, Figure 3). The patients with infarct-pattern scar had a significantly lower global stress MBF than those with non-ischemic scar only or no scar (infarct vs. non-ischemic vs. no LGE; 2.04 ± 0.48 vs. 2.54 ± 0.65 vs. 2.75 ± 0.87 ml/g/min, *p* = *0.003;* Figure 4). There was no difference in the global blood flow between patients with a non-ischemic scar and no scar (*p* = *0.26)*. The MPR was lower in the COVID cohort than in the matched controls (2.67 ± 0.87 vs. 2.95 ± 1.03 ml/g/min, *p* = *0.049*) driven by the higher resting MBF in the COVID cohort (0.99 ± 0.34 vs.0.89 ± 0.24 ml/g/min, *p* = *0.02*), but the values were in line with the normal values in the studies using the same quantitative perfusion methodology (9).

**Table 2:** Results.

**Characteristic**	**COVID** ***n* = 90^**a**^**	**Control** ***n* = 90^**a**^**	**HV** ***n* = 27^**a**^**	***p*-value^**b**^ (COVID vs. control)**	***p*-value^**b**^** **(All groups)**
Global Stress MBF (ml/g/min)	2.53 (0.77)	2.52 (0.79)	3.00 (0.76)	0.93	**0.012**
Global Rest MBF (ml/g/min)	0.99 (0.34)	0.89 (0.24)	0.86 (0.26)	**0.018**	**0.022**
MPR	2.67 (0.87)	2.95 (1.03)	3.63 (0.75)	**0.049**	**<0.001**

a *Statistics presented: Mean (SD); n (%)*.

b *Statistical tests performed: One-way ANOVA; Fisher's exact test. p-values reaching statistical significance (p < 0.05) are highlighted in bold*.

##### Predictors of Global Stress MBF

The multivariable predictors of low global stress MBF were age (OR.90, 95%CI 0.82–0.98, *p* =*0.02*), male sex (OR 0.60, 95% CI 0.46–0.78, *p* = < *0.001*), and history of hypertension (OR 1.52, 95%CI 1.23–1.89, *p* = < *0.001*). Prior COVID-19 illness was not associated with lower global stress MBF (OR 1.1, 95%CI 0.89–1.35, *p* = *0.4*) (Table 3).

**Table 3:** Multivariable analysis of global stress myocardial blood flow in the COVID and propensity-matched control cohorts.

	**Full model**			**Final model**		
**Characteristic**	**OR**	**95% CI^**a**^**	***p*-value**	**OR**	**95% CI^**a**^**	***p*-value**
COVID status	1.10	0.85, 1.41	0.5	1.10	0.89, 1.35	0.4
Age^*^	0.90	0.82, 0.99	0.027	0.90	0.82, 0.98	**0.019**
Male Sex	0.60	0.47, 0.79	<0.001	0.60	0.46, 0.78	**<0.001**
Type 2 Diabetes	0.81	0.64, 1.02	0.071	0.80	0.64, 1.00	0.057
Hypertension	1.50	1.20, 1.87	<0.001	1.52	1.23, 1.89	**<0.001**
Hypercholesterolemia	1.09	0.88, 1.35	0.4			
Infarct pattern LGE	0.70	0.47, 1.06	0.091	0.71	0.48, 1.03	0.074
Non-ischemic LGE	0.97	0.71, 1.33	0.9			

a *CI, Confidence Interval*.

* *Scaled by epochs of 10 years. p-values reaching statistical significance (p < 0.05) are highlighted in bold*.

##### Regional Stress Perfusion

Thirty-two patients had localized segmental stress perfusion defects (median 3 [IQR 3-7] segments). Of those with perfusion defects, only six out of 32 had perfusion defects solely matched to a region of infarct-pattern LGE, whereas 26 out of 32 patients had unmatched perfusion defects (Figures 2, 4).

## Discussion

In this multicenter study of COVID-19 survivors, we demonstrated that at 2 months after severe, hospitalized infection, the global stress MBF is comparable to the propensity-matched controls with proven unobstructed coronaries and no scar. However, over half of the COVID patients have evidence of either infarct or myocarditis-like scar, and almost a third had evidence of regional ischemia, suggestive of occult coronary artery disease, including a quarter of those with an otherwise normal CMR. While microvascular thrombosis may also play a role, there is little evidence here to suggest a significant impact on the global myocardial perfusion in surviving patients.

Myocardial damage during acute COVID-19 illness predicts the severity (18) and outcomes of acute infection, with CMR scans demonstrating infarction and inflammation in convalescent patients (12). In accordance with previous publications, the prevalence of LGE in this cohort was high, but the overall burden and functional impact were relatively low (19). However, the effects of COVID-19 on myocardial perfusion have so far remained incompletely characterized. We demonstrated that stress myocardial blood flow after severe COVID-19 shows no difference to risk factor matched controls; importantly stress blood flow was not predicted by COVID status but by common risk factors for coronary artery disease.

While MPR was found to be slightly reduced in the COVID cohort compared with the controls, this was driven primarily by a higher resting MBF. In a recent study by Drakos et al., there was a significant reduction in MPR in *n* = 22 patients with persistent symptoms post-COVID-19 when assessed by coronary sinus flow, driven by both a lower stress MBF and higher resting MBF (11). This study had several methodological and technological differences to this study (assessment of MBF by coronary sinus flow, unmatched cohorts, inclusion of patients with persistent symptoms only, and exclusion of severe COVID-19). There are alternative explanations for higher resting MBF in our cohort compared with the controls (which may include differences in heart rate and medications between cohorts), thus we do not believe that these small borderline significant differences provide convincing evidence of microvascular dysfunction.

The finding of inducible ischemia in almost a third of the patients suggests that the putative mechanism of troponin elevation reflected a supply-demand-mismatch, whether epicardial or microvascular (20), representing “a COVID stress test”. It is important, however, to acknowledge the multiple mechanisms of troponin elevation in the context of severe illness (21). We hypothesize that the high burden of infarct-pattern LGE was pre-existing, and driven more by the demographics of included patients (older, high incidence of cardiovascular risk factors), than by the COVID-19 illness, especially given the relatively modest troponin rise seen. This is particularly relevant as our sample reflects a relatively sick group of patients (all admitted, 44% to ICU; 85 out of 90 with elevated troponin). The prevalence of the infarct pattern scar is similar to the previous studies which cited a rate of 17% of occult MI by CMR in a cohort with a similar cardiovascular risk profile. Our group has previously shown the rate of infarct pattern scar in a contemporary chest pain cohort referred for adenosine-stress CMR to be as high as 29.5% (22, 23). Cardiovascular magnetic resonance is the most suited non-invasive modality for the assessment of unexplained troponin rise, as it can confirm the presence, type, and extent of myocardial injury. Interestingly, nearly half of the troponin-positive patients (39/85) had no evidence of scar on CMR. This suggests that while troponin leak is associated with poor prognosis in acute illnesses, in many cases it is not followed by medium-term structural or functional effects, including sensitive LGE imaging or global MBF quantification.

Autopsy studies of COVID-19 patients have demonstrated microvascular thrombosis in the lungs and heart (7, 24, 25). In the cardiac studies, microthrombi were identified as the cause of myocyte necrosis in 64% of patients after COVID-19 related death. Other studies have also identified these in intra-myocardial small vessels (26, 27). Many of these thrombi were not associated with any significant myocardial injury. It is plausible that microthrombisis may lead to exceptionally localized myocyte injury. Although this study was designed specifically to take advantage of identical scanner platforms and access to state-of-the-art quantitative myocardial perfusion and myocardial tissue characterization CMR technology [supported by MRI phantom quality controlled analysis (22)], very localized myocyte injury may be undetectable by the current CMR technologies (LGE or T1/T2/ECV/MBF mapping) or may have resolved without detectable damage in convalescent patients. This entity is potentially somewhat distinct from what would conventionally be referred to as a “microvascular disease”, which is commonly perceived as a global phenomenon in response to a systemic disease process (type 2 diabetes, for example), without demonstrable inducible perfusion defects or regional wall motion abnormalities (28).

Microvascular disease has also been shown to have regionality and may have a subendocardial distribution (29). The regional perfusion defects found in our study could be due to regional microvascular disease if the epicardial coronaries are found unobstructed. A normal global stress MBF in troponin-positive or persistently symptomatic individuals is reassuring to an extent but may not have the sensitivity to resolve regional microvascular complications.

Overall, multi-parametric stress perfusion CMR in convalescent post-COVID-19 patients identified previously unsuspected inducible ischemia, myocardial infarction, and myocarditis-pattern non-ischemic scar. The addition of stress perfusion imaging offers incremental information by the detection of regional ischemia in the absence of a scar. There is little evidence of a reduction in global perfusion after severe COVID-19 compared to matched controls, which may reflect the limited impact of microvascular thrombosis on overall perfusion in surviving patients even if it causes persistent regional differences in myocardial blood flow.

## Limitations

This study reports findings in a group of patients who survived COVID-19 infection and is therefore affected by survivor bias. Patients with contraindications to CMR were excluded, though this is a small minority of the patients. We have characterized the spectrum of myocardial injury and perfusion abnormalities in recovered, predominantly severe, COVID-19 illness that persists to a median of 2 months after the infection (community and mild disease were not investigated). This study does not offer insights into myocardial blood flow, structure or function during the infection or in the immediate post-infective period, nor can it, without longitudinal data, determine if abnormalities detected evolve or regress over time. The wide time interval between the admission episode and CMR further compounds this. Data on persistent symptoms at the time of CMR was not available for the majority of patients, thus we cannot draw conclusions pertaining to the association of these with MBF. Furthermore, without anatomical data, we cannot confirm the presence and cause (epicardial vs. microvascular) of perfusion abnormalities. However, this study yields pertinent preliminary data which may have important clinical implications for patients. Further granularity will be determined with future ongoing United Kingdom and international studies, some of which will acquire or capture coronary anatomical data [COVID-HEART [ISRCTN58667920]; CISCO-19 (30)].

## Conclusion

This multicenter cohort of severe hospitalized COVID-19 infection identified no difference in the global stress MBF in COVID-19 survivors compared with the risk factor matched controls, but regional perfusion defects are common. Overall, the findings are reassuring that COVID-19 is unlikely to result in gross and persistent global microvascular phenomena. The high burden of regional ischemia may be due to regional microvascular disease but is more likely due to pre-existing coronary disease, but neither can be proven in the absence of anatomical imaging. This should therefore be considered hypothesis-generating for future studies.

## Data Availability Statement

The raw data supporting the conclusions of this article will be made available by the authors, without undue reservation.

## Ethics Statement

The studies involving human participants were reviewed and approved by the West Midlands – Edgbaston Research Ethics Committee for the use of patient's clinical data for research purposes (RFH and Imperial sites; REC reference 20/WM/0208) and from the Joint University College London/University College London Hospitals Research Ethics Committee (UCLH site; REC reference 07/H0715/101). The patients/participants provided their written informed consent to participate in this study.

## Author Contributions

GT: concept and design, writing for publication, revision of drafts, and submission of manuscript. TT: concept and design, data analysis, writing of manuscript, revision, and approval of final submission. MF, PK, GC, CM, RB, HX, RD, ASe, and DK: revision and approval of submission. JM, TK, KV, JB, LC, DA, SY, HK, JA, KK, ASe, and ASh: data analysis, revision of drafts, and approval of submission. List ASh with others who contributed data analysis, revision of drafts, and approval of submission. All authors listed have made a substantial, direct, and intellectual contribution to the work and approved it for publication.

## Funding

GT was supported by a British Heart Foundation (BHF) Clinical Research Training Fellowship (FS/CRTF/21/24128). DK and RB were supported by the National Institute for Health Research (NIHR) University College London Hospitals (UCLH) Biomedical Research Center. JM was directly supported by the (UCLH) and Barts NIHR Biomedical Research Centers and through a BHF Accelerator Award. MF and TT (ICRF #34374) are funded by a British Heart Foundation (BHF) Intermediate Fellowship.

## Conflict of Interest

The authors declare that the research was conducted in the absence of any commercial or financial relationships that could be construed as a potential conflict of interest.

## Publisher's Note

All claims expressed in this article are solely those of the authors and do not necessarily represent those of their affiliated organizations, or those of the publisher, the editors and the reviewers. Any product that may be evaluated in this article, or claim that may be made by its manufacturer, is not guaranteed or endorsed by the publisher.

## Supplementary Material

Supplementary Table 1 Table showing the covariate balance before and after propensity matching. The Data are presented as means and standardized mean differences.

Supplementary Figure 1 CMR imaging protocol for the study.

Supplementary Figure 2 Perfusion Mapping Quality Assurance and Outputs.

Supplementary Figure 3 CONSORT diagram describing the pathway from the clinical CMR post-COVID to the final number of patients who underwent stress CMR.

## Abbreviations

CMR: cardiovascular magnetic resonance
COVID-19: coronavirus disease 2019
ICU: intensive care unit
LGE: late gadolinium enhancement
sMBF: stress myocardial blood flow
LV: left ventricular
MI: myocardial infarction
MOLLI: modified look-locker inversion recovery
SARS-CoV-2: severe acute respiratory syndrome coronavirus 2.

## References

[1] Chapman AR Bularga A Mills NL. High-sensitivity cardiac troponin can be an ally in the fight against COVID-19. Circulation. (2020) 141:1733–5. 10.1161/CIRCULATIONAHA.120.047008 32251612

[2] Bonow RO Fonarow GC O'Gara PT Yancy CW. Association of coronavirus disease 2019 (COVID-19) with myocardial injury and mortality. JAMA Cardiology. (2020) 5:751. 10.1001/jamacardio.2020.1105 32219362

[3] Guo T Fan Y Chen M Wu X Zhang L He T. Cardiovascular implications of fatal outcomes of patients with coronavirus disease 2019 (COVID-19). JAMA Cardiology. (2020) 5:811. 10.1001/jamacardio.2020.1017 32219356 PMC7101506

[4] Montone RA Iannaccone G Meucci MC Gurgoglione F Niccoli G. Myocardial and microvascular injury due to coronavirus disease 2019. European Cardiology Review. (2020) 15:e52. 10.15420/ecr.2020.22 32617121 PMC7325215

[5] Lowenstein CJ Solomon SD. Severe COVID-19 Is a microvascular disease. Circulation. (2020) 142:1609–11. 10.1161/CIRCULATIONAHA.120.050354 32877231 PMC7580651

[6] Ince C Mayeux PR Nguyen T Gomez H Kellum JA Ospina-Tascón GA. The endothelium in sepsis. Shock. (2016) 45:259–70. 10.1097/SHK.0000000000000473 26871664 PMC5281063

[7] Pellegrini D Kawakami R Guagliumi G Sakamoto A Kawai K Gianatti A. Microthrombi as a major cause of cardiac injury in COVID-19: a pathologic study. Circulation. (2021) 143:1031–42. 10.1161/CIRCULATIONAHA.121.055585 33480806

[8] Kellman P Hansen MS Nielles-Vallespin S Nickander J Themudo R Ugander M. Myocardial perfusion cardiovascular magnetic resonance: optimized dual sequence and reconstruction for quantification. Journal of Cardiovascular Magnetic Resonance. (2017) 19:43. 10.1186/s12968-017-0355-5 28385161 PMC5383963

[9] Kotecha T Martinez-Naharro A Boldrini M Knight D Hawkins P Kalra S. Automated pixel-wise quantitative myocardial perfusion mapping by CMR to detect obstructive coronary artery disease and coronary microvascular dysfunction: validation against invasive coronary physiology. JACC Cardiovasc Imaging. (2019) 12:1958–69. 10.1016/j.jcmg.2018.12.022 30772231 PMC8414332

[10] Engblom H Xue H Akil S Carlsson M Hindorf C Oddstig J. Fully quantitative cardiovascular magnetic resonance myocardial perfusion ready for clinical use: a comparison between cardiovascular magnetic resonance imaging and positron emission tomography. J Cardiovasc Magn Reson. (2017) 19:78. 10.1186/s12968-017-0388-9 29047385 PMC5648469

[11] Drakos S Chatzantonis G Bietenbeck M Evers G Schulze AB Mohr M. A cardiovascular magnetic resonance imaging-based pilot study to assess coronary microvascular disease in COVID-19 patients. Scientific Reports. (2021) 11:15667. 10.1038/s41598-021-95277-z 34341436 PMC8329060

[12] Kotecha T Knight DS Razvi Y Kumar K Vimalesvaran K Thornton G. Patterns of myocardial injury in recovered troponin-positive COVID-19 patients assessed by cardiovascular magnetic resonance. European Heart Journal. (2021) 1866–1878. 10.3410/f.739571449.793583038 33596594 PMC7928984

[13] Han Y Chen T Bryant J Bucciarelli-Ducci C Dyke C Elliott MD. Society for cardiovascular magnetic resonance (SCMR) guidance for the practice of cardiovascular magnetic resonance during the COVID-19 pandemic. J Cardiovasc Magn Reson. (2020) 22:26. 10.1186/s12968-020-00628-w 32340614 PMC7184243

[14] Kellman P Hansen MS. T1-mapping in the heart: accuracy and precision. Journal of Cardiovascular Magnetic Resonance. (2014) 16:2. 10.1186/1532-429X-16-2 24387626 PMC3927683

[15] Giri S Chung YC Merchant A Mihai G Rajagopalan S Raman SV. T2 quantification for improved detection of myocardial edema. J Cardiovasc Magn Reson. (2009) 11:56. 10.1186/1532-429X-11-56 20042111 PMC2809052

[16] Kellman P Arai AE. Cardiac imaging techniques for physicians: late enhancement. J Magn Reson Imaging. (2012) 36:529–42. 10.1002/jmri.23605 22903654 PMC3428749

[17] Xue H Davies RH Brown LAE Knott KD Kotecha T Fontana M. Automated inline analysis of myocardial perfusion MRI with deep learning. Radiology: Artificial Intelligence. (2020) 2:e200009. 10.1148/ryai.2020200009 33330849 PMC7706884

[18] Sabatino J De Rosa S Di Salvo G Indolfi C. Impact of cardiovascular risk profile on COVID-19 outcome. A meta-analysis. PLoS ONE. (2020) 15:e0237131. 10.1371/journal.pone.0237131 32797054 PMC7428172

[19] Kim JY Han K Suh YJ. Prevalence of abnormal cardiovascular magnetic resonance findings in recovered patients from COVID-19: a systematic review and meta-analysis. Journal of Cardiovascular Magnetic Resonance. (2021) 23:100. 10.1186/s12968-021-00792-7 34479603 PMC8414035

[20] Chen T Wu D Chen H Yan W Yang D Chen G. Clinical characteristics of 113 deceased patients with coronavirus disease 2019: retrospective study. BMJ. (2020) 368:m1091. 10.1136/bmj.m1091 32217556 PMC7190011

[21] Bansal M. Cardiovascular disease and COVID-19. Diabetes & Metabolic Syndrome: Clinical Research & Reviews. (2020) 14:247–50. 10.1016/j.dsx.2020.03.013 32247212 PMC7102662

[22] Knott KD Seraphim A Augusto JB Xue H Chacko L Aung N. The prognostic significance of quantitative myocardial perfusion: an artificial intelligence based approach using perfusion mapping. Circulation. (2020) 141:1282–91. 10.1161/CIRCULATIONAHA.119.044666 32078380 PMC7176346

[23] Schelbert EB Cao JJ Sigurdsson S Aspelund T Kellman P Aletras AH. Prevalence and prognosis of unrecognized myocardial infarction determined by cardiac magnetic resonance in older adults. JAMA. (2012) 308:890. 10.1001/2012.jama.11089 22948699 PMC4137910

[24] Ackermann M Verleden SE Kuehnel M Haverich A Welte T Laenger F. Pulmonary vascular endothelialitis, thrombosis, and angiogenesis in Covid-19. New England Journal of Medicine. (2020) 383:120–8. 10.1056/NEJMoa2015432 32437596 PMC7412750

[25] Magro C Mulvey JJ Berlin D Nuovo G Salvatore S Harp J. Complement associated microvascular injury and thrombosis in the pathogenesis of severe COVID-19 infection: A report of five cases. Translational Research. (2020) 220:1–13. 10.1016/j.trsl.2020.04.007 32299776 PMC7158248

[26] Bois MC Boire NA Layman AJ Aubry M-C Alexander MP Roden AC. COVID-19–associated nonocclusive fibrin microthrombi in the heart. Circulation. (2021) 143:230–43. 10.1161/CIRCULATIONAHA.120.050754 33197204 PMC7805556

[27] Elsoukkary S Mostyka SM Dillard A Berman R Ma DX Chadburn LA. Steven, autopsy findings in 32 patients with COVID-19: a single-institution experience. Pathobiology. (2021) 88:56–68. 10.1159/000511325 32942274 PMC7573917

[28] Panza JA Laurienzo JM Curiel RV Unger EF Quyyumi AA Dilsizian V. Investigation of the mechanism of chest pain in patients with angiographically normal coronary arteries using transesophageal dobutamine stress echocardiography. Journal of the American College of Cardiology. (1997) 29:293–301. 10.1016/S0735-1097(96)00481-0 9014980

[29] Rahman H Scannell CM Demir OM Ryan M Mcconkey H Ellis H. High-resolution cardiac magnetic resonance imaging techniques for the identification of coronary microvascular dysfunction. JACC: Cardiovascular Imaging. (2020) 14:987–98. 10.1016/j.jcmg.2020.10.015 33248969

[30] Mangion K Morrow A Bagot C Bayes H Blyth KG Church C. The chief scientist office cardiovascular and pulmonary imaging in SARS coronavirus disease-19 (CISCO-19) study. Cardiovasc Res. (2020) 116:2185–96. 10.1093/cvr/cvaa209 32702087 PMC7454350

