# Supplementary material for: Myocardial Perfusion Imaging After Severe COVID-19 Infection Demonstrates Regional Ischemia Rather Than Global Blood Flow Reduction
Source: Front Cardiovasc Med. 2021 Dec 7;8:764599. doi: 10.3389/fcvm.2021.764599 (PMC8688537; doi:10.3389/fcvm.2021.764599)

**SUPPLEMENTARY DATA**

**Table S1: Propensity Matching**

| **Summary of Balance for Unmatched Data:** | | |  |
| --- | --- | --- | --- |
|  | **Means COVID** | **Means Control** | **Std. Mean Diff.** |
| **Age** | 61.43 | 58.36 | 0.25 |
| **Sex** | 0.83 | 0.48 | 0.95 |
| **Hypertension** | 0.52 | 0.45 | 0.15 |
| **Type 2 Diabetes** | 0.32 | 0.25 | 0.16 |
| **Smoker** | 0.72 | 0.72 | 0.004 |
| **Summary of Balance for Matched Data:** | | |  |
|  | **Means COVID** | **Means Control** | **Std. Mean Diff.** |
| **Age** | 61.43 | 58.91 | 0.21 |
| **Sex** | 0.83 | 0.81 | 0.06 |
| **Hypertension** | 0.52 | 0.47 | 0.11 |
| **Type 2 Diabetes** | 0.32 | 0.29 | 0.07 |
| **Smoker** | 0.72 | 0.70 | 0.05 |

**Figure S1 CMR Protocol**


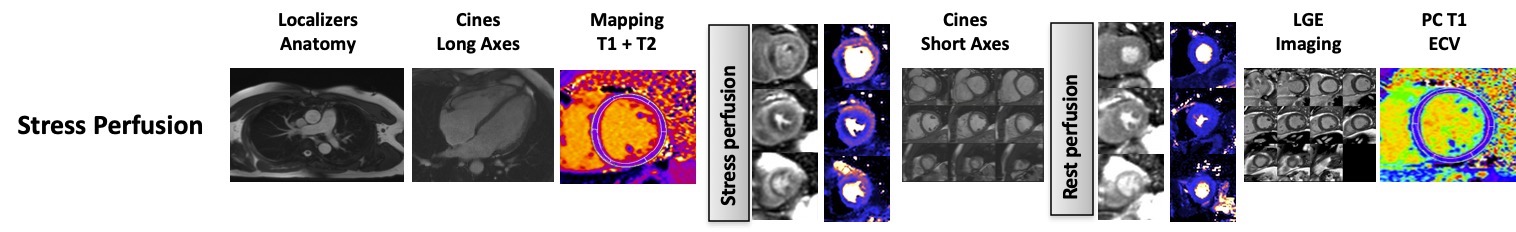


**Figure S2 Perfusion Mapping Quality Assurance and Outputs**

**
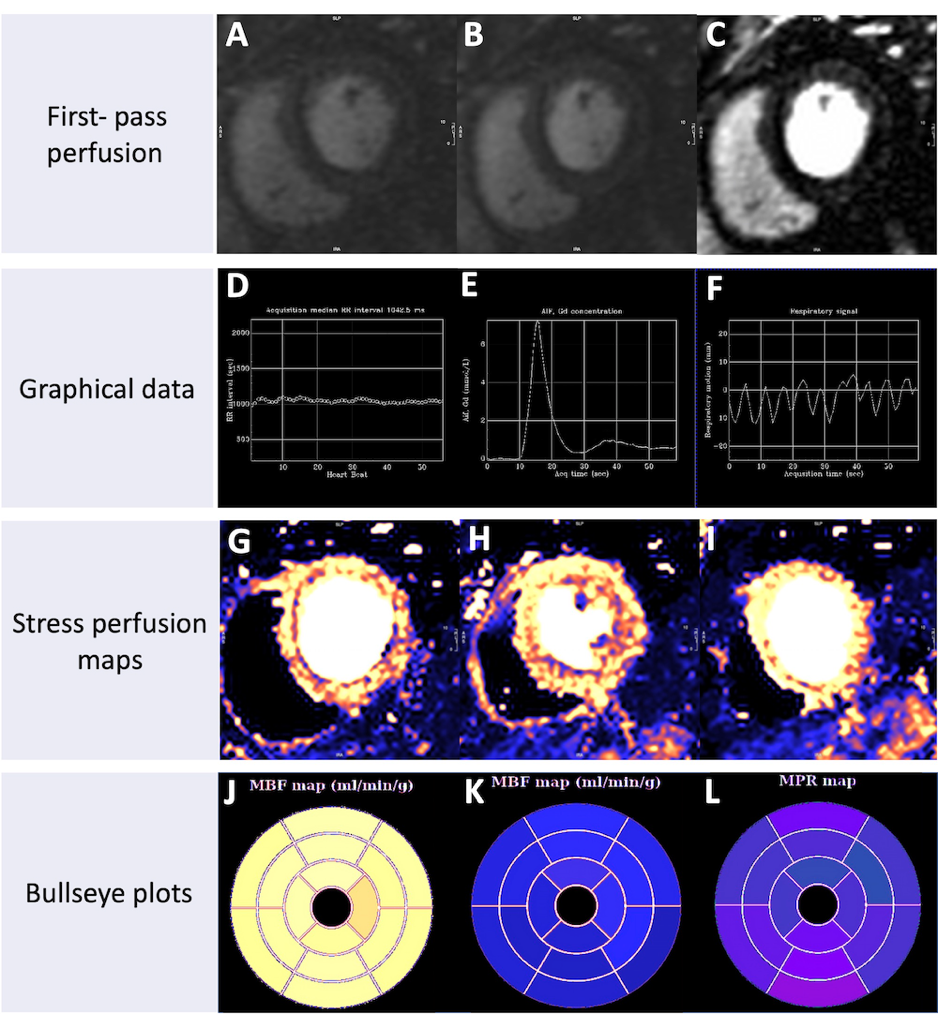
**

**First-pass perfusion A.** Raw data **B.** Motion corrected (MOCO) image **C.** Gadolinium corrected image

**Graphical data D.** Plot of RR intervals **E.** Arterial Input Function **F.** Respiratory motion

**Stress perfusion maps** **G.** Base **H.** Mid **I.** Apex

**Bullseye plots J.** Stress myocardial blood flow (MBF) **K.** Rest MBF **L.** Myocardial perfusion reserve (MPR)

**Supplementary Figure 3 CONSORT diagram**

**Figure S3.** CONSORT diagram describing the pathway from clinical CMR post-COVID to final number of patients who underwent stress CMR. Abbreviations - CMR (cardiac magnetic resonance imaging)


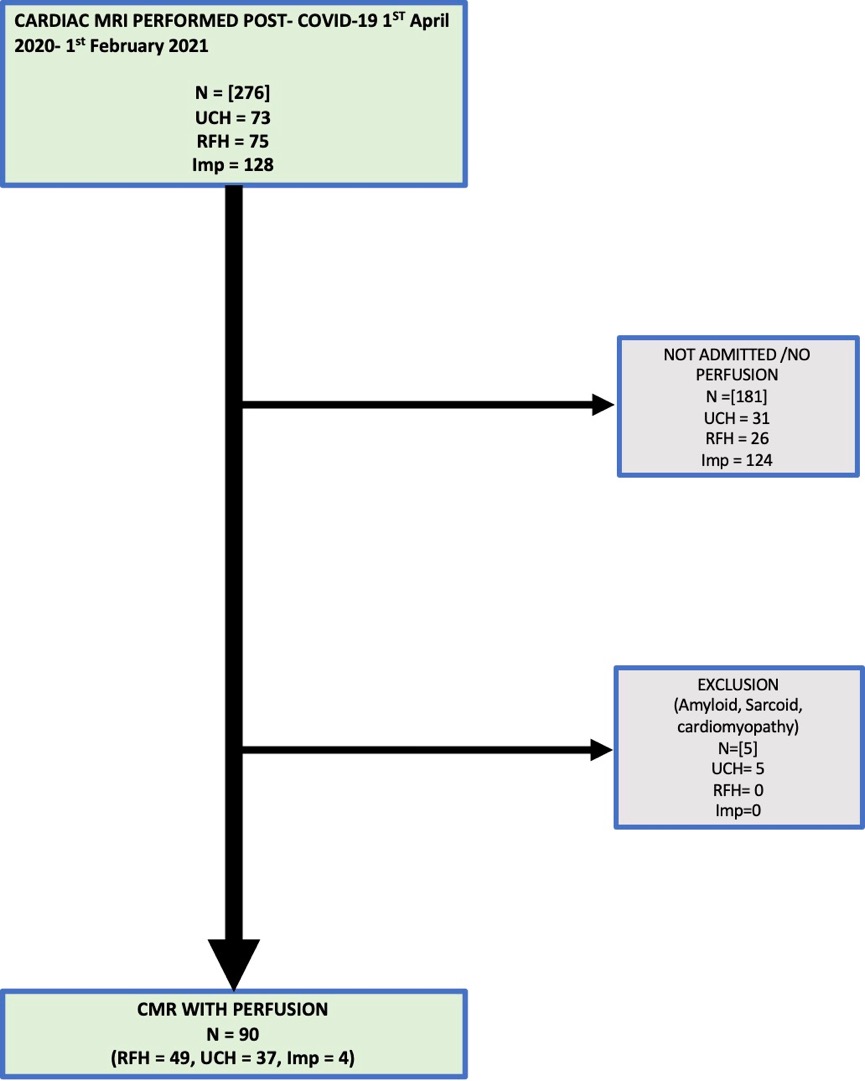

Supplement: Supplementary Table 1 — Table showing the covariate balance before and after propensity matching. The Data are presented as means and standardized mean differences. [file Data_Sheet_1.docx]
